# CD44 glycoprotein in cancer: a molecular conundrum hampering clinical applications

**DOI:** 10.1186/s12014-018-9198-9

**Published:** 2018-06-27

**Authors:** Rita Azevedo, Cristiana Gaiteiro, Andreia Peixoto, Marta Relvas-Santos, Luís Lima, Lúcio Lara Santos, José Alexandre Ferreira

**Affiliations:** 1 Experimental Pathology and Therapeutics Group, Portuguese Institute of Oncology, Rua Dr. António Bernardino de Almeida, 4200-072 Porto, Portugal; 2 Institute of Biomedical Sciences Abel Salazar, University of Porto, Porto, Portugal; 3 Program of Immunology and Immunotherapy, Center for Applied Medical Research (CIMA), Pamplona, Spain; 4 Instituto de Investigação e Inovação em Saúde (I3S), Universidade do Porto, Porto, Portugal; 5 Porto Comprehensive Cancer Center (P.ccc), Porto, Portugal; 6 University Fernando Pessoa, Porto, Portugal; 7 Glycobiology in Cancer, Institute of Molecular Pathology and Immunology of the University of Porto (IPATIMUP), Porto, Portugal; 8 International Iberian Nanotechnology Laboratory (INL), Braga, Portugal

**Keywords:** CD44, Glycosylation, Cancer biomarkers, Nomenclature, CD44 isoforms

## Abstract

CD44 is a heavily glycosylated membrane receptor playing a key role in cell adhesion, signal transduction and cytoskeleton remodelling. It is also one of the most studied glycoproteins in cancer, frequently explored for stem cell identification, and associated with chemoresistance and metastasis. However, CD44 is a general designation for a large family of splicing variants exhibiting different degrees of glycosylation and, potentially, functionally distinct roles. Moreover, structural diversity associated with ambiguous nomenclature has delayed clinical developments. Herein, we attempt to comprehensively address these aspects and systematize CD44 nomenclature, setting milestones for biomarker discovery. In addition, we support that CD44 may be an important source of cancer neoantigens, most likely resulting from altered splicing and/or glycosylation. The discovery of potentially targetable CD44 (glyco)isoforms will require the combination of glycomics with proteogenomics approaches, exploring customized protein sequence databases generated using genomics and transcriptomics. Nevertheless, the necessary high-throughput analytical and bioinformatics tools are now available to address CD44 role in health and disease.

The transmembrane glycoprotein receptor CD44 plays a key role in cell adhesion to the extracellular matrix and interacts with growth factors and several extracellular ligands, including hyaluronic acid, collagen, osteopontin and many metalloproteinases to drive signal transduction and cytoskeleton rearrangements [1]. By interacting with co-factors and adaptor proteins, CD44 has been further implicated in lymphocyte homing, haematopoiesis, cell migration and adhesion, tumour invasion and metastasis [1]. Several isoforms of CD44 can be generated through the insertion of alternative exons at the variable region in a process regulated at both tissue and cellular levels. While ubiquitously expressed in healthy adult and foetal tissues, the molecular plasticity of alternatively spliced CD44 accounts for diversified functional roles. However, the intricate correlation between CD44 isoforms and underlying biological functions is yet to be fully disclosed. It has been long described that malignant transformation and progression are accompanied by a deregulation of CD44 splicing mechanisms, comprehensively addressed in recent reviews [2, 3]. The events leading to CD44 isoform molecular remodelling have direct implications in several cancer hallmarks and appear to vary according to the type of lesion, supporting the existence of disease-specific molecular fingerprints and potentially targetable biomarkers [4]. Not surprisingly, CD44 has been a hot topic in cancer research, frequently associated with more aggressive phenotypes and widely explored for cancer stem-cell identification [5]. Particular focus has been set on narrowing CD44 screening to its cancer-associated isoforms envisaging the necessary sensitivity and specificity for clinical applications. However, the lack of protocols for its full isoform discrimination at the protein level, and the existence of many functionally distinct isoforms poses a major drawback. Currently, these hurdles can only be partially circumvented by targeting CD44 transcripts using variant-specific probes or by emerging RNAseq approaches. Analytical difficulties are aggravated by vast post-translational modifications, with emphasis on the very high glycosylation density of variable regions. Moreover, glycans often present a non-templated and context-dependent nature, with several glycoforms coexisting for the same protein on a given biological milieu, further increasing CD44 molecular and functional diversity (Table 1). This poses a major challenge for identification by conventional immunoassays as well as high-throughput proteomics, which has delayed the definition of CD44 isoforms in health and disease.

**Table 1:** Proposed CD44 nomenclature for experimentally observed isoforms, its correspondence with UniProt and NCBI databases and predicted *N*- and *O*-glycosylation sites

Proposed nomenclature	UniProt	NCBI	Predicted glycosylation sites		
			*N*-glycosylation^a^	*O*-glycosylation^b^	Total
CD44v2-10	Isoform 1	Isoform 1	8	146	154
CD44v3-10	Isoform 4	Isoform 2	8	133	141
CD44v8-10	Isoform 10	Isoform 3	7	79	86
CD44v10	Isoform 11	Isoform 6	7	56	63
CD44s	Isoform 12	Isoform 4	6	32	38
CD44st	Isoform 15	Isoform 8	6	32	38
CD44s-exon 15	Isoform 18	Isoform 7	6	23	29
CD44soluble	Isoform 19	Isoform 5	2	3	5

^a^N-glycosylation sites predicted using NetNGlyc server 1.0 (http://www.cbs.dtu.dk/services/NetNGlyc/)

^b^O-glycosylation sites predicted using NetOGlyc server 4.0 (http://www.cbs.dtu.dk/services/NetOGlyc/)

The absence of nomenclature standardization also emerges as a key issue, making inter-study comparisons and clinical translation almost impossible. Showcasing some examples, CD44H and CD44E terminologies arise from the first observations in hematopoietic and epithelial cells; gp116 and gp85 distinguish glycoforms by molecular weights (116 and 85 kDa, respectively); CD44 hematopoietic cell E-/L-selectin ligand (HCELL) refers to CD44 isoforms expressed in hematopoietic and cancer cells showing elevated sialofucosylated glycans content and high affinity for E-/L-selectin ligands [6, 7]. All these designations fail to provide clear insights on the molecular nature of the isoforms. Notwithstanding, some studies adopt a nomenclature based on commercial names of the used monoclonal antibodies, highlighting the targeted variable exon, being CD44v3, CD44v6, CD44v9 amongst the most associated with cancer, including chemoresistance and prognosis [8, 9]. Moreover, these studies often disregard that the analysis of a specific variable exon can result in the detection of all isoforms containing it instead of one particular protein [10]. In addition, most variable regions may be significantly hindered by dense glycosylation, biasing detection. On the other hand, reports exploring the transcriptome have emerged as a powerful tool for determining CD44 isoforms diversity; however, few have provided the necessary validation at the protein level due to above mentioned analytical difficulties. Another important source of nomenclature ambiguity results from the direct translation of results from *Mus musculus* studies to *Homo sapiens* disregarding that mice CD44 gene presents an extra variable exon (v1) not present in humans. Further contributing to ambivalence, UniProt and NCBI protein databases adopt different designations for the same isoform (illustrated in Table 1). Altogether these aspects impact negatively on our understanding of the biological and clinical relevance of CD44 isoforms in cancer and other diseases, urging nomenclature standardization.

Facing these challenges, we have conducted a comprehensive in silico analysis of CD44 isoforms through NCBI and UniProt databases, using BLAST and TMHMM Server v2.0 tools. The human *CD44* gene is located on the short arm of chromosome 11 [GRCh38.p7, NC_000011.10 (35138870-35232402)] and its precursor mRNA consists of 19 exons. Namely, exons 1–16 encode the extracellular domain, exon 17 encodes the highly conserved transmembrane domain, and exons 18 and 19 encode the cytoplasmic domain. In silico analysis in NCBI has predicted over twenty-one possible mRNA transcripts derived from the alternative splicing of exons 6–14 and 18, eleven of which were also found in UniProt. However, only eight have been experimentally confirmed (detailed in Table 1 and Fig. 1), specifically six isoforms with variable extracellular domain extensions, one isoform with a truncated cytoplasmic tail, and one isoform truncated at the extracellular domain. Here we attempt to standardize the nomenclature for the above described isoforms through a logical terminology. Following pre-existing designations, we propose that human CD44 isoforms originated by alternative splicing of the variable region should highlight the included exons. Conversely, isoforms lacking the variable region should present a nominal designation. Figure 1 constitutes a schematic representation of the experimentally determined human CD44 isoforms, and the proposed nomenclature is described below:

**Fig. 1:**
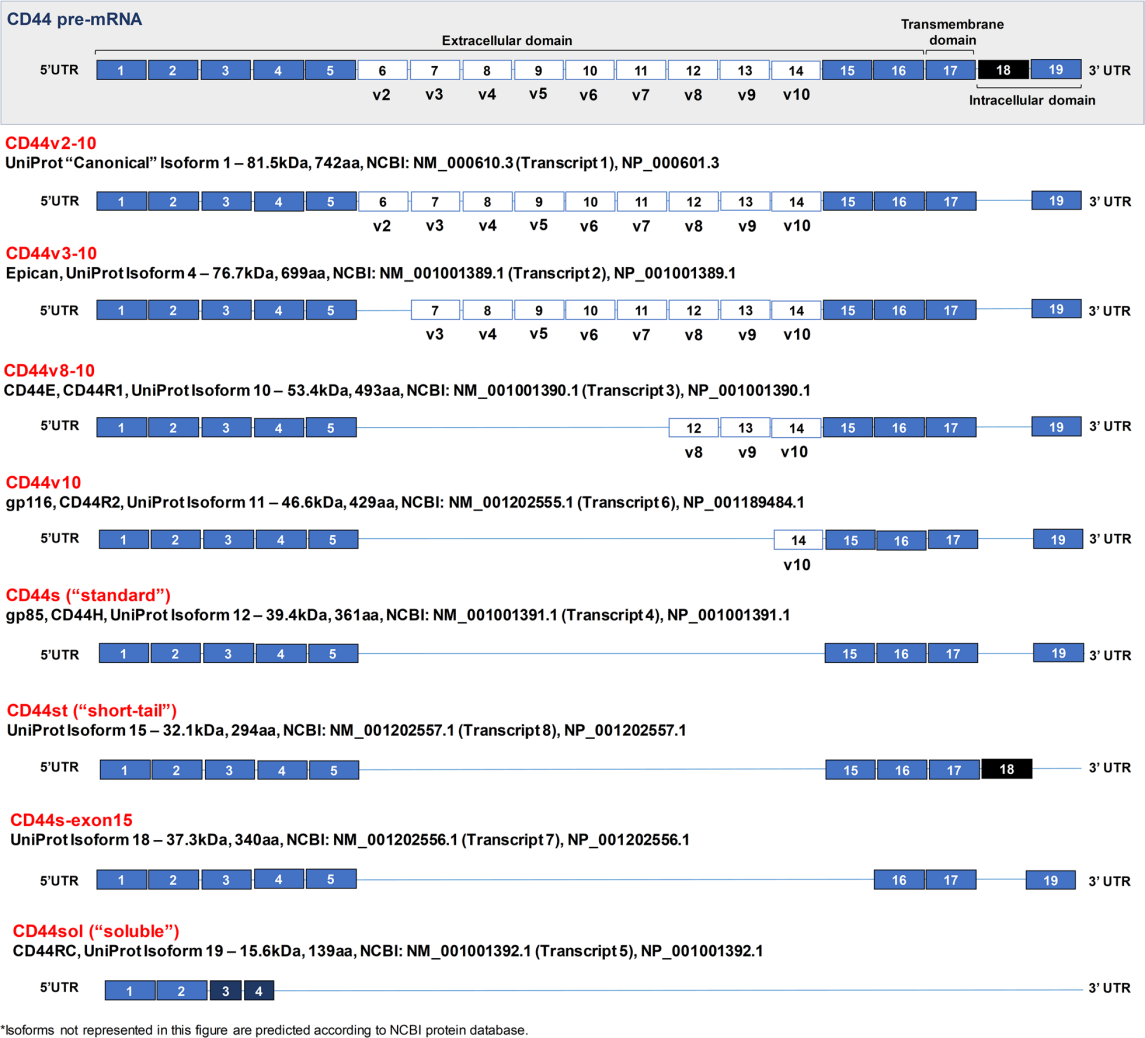
Schematic representation of experimentally confirmed human CD44 pre-mRNA and respective isoforms. Blue filled boxes represent constant region exons, while white filled boxes represent exons of the variable region present in the designated CD44 isoform. Dark blue filled boxes with reduced box size represent truncated exons from the constant region. The blue line represents missing exon(s). Exon 18, filled black, contains an early 3’UTR and only makes part of CD44st isoform

- **CD44v2-10** The canonical CD44 isoform includes a peptide sequence encoded by exons 6–14, while splicing out exon 18. This isoform has a predicted molecular weight of 82 kDa but presents extensive glycosylation, thereby arising to approximately 250 kDa or higher.
- **CD44v3-10** Also known as epican, this isoform results from the retention of exons 7–14 (v3–v10) and splicing out of exon 18. Its unmodified form has 77 kDa, resulting in an up to 200 kDa glycoprotein after post-translational modifications.
- **CD44v8-10** Also known as CD44E or CD44R1, this isoform is originated through retention of exons 12–14 (v8–v10) and splicing out of exon 18. It has 53 kDa in its unaltered form, while reaching 130 kDa in its glycosylated form.
- **CD44v10** Also known as gp116 or CD44R2, this isoform retains the variable exon 14 (v10), while splicing out exon 18. The unglycosylated form has approximately 47 kDa whereas the glycosylated forms have been observed at 120 kDa.
- **CD44s** Also known as CD44H or gp85, the standard form of CD44 splices out all variable exons and exon 18. Originally with 39 kDa, the subsequent post-translational addition of *N*-linked and *O*-linked oligosaccharides gives rise to a 85–90 kDa glycoprotein.
- **CD44st** Also known as short-tail or tail-less, is a 32 kDa isoform splicing out the variable region and exon 19, while retaining exon 18. Importantly, exon 18 contains a stop codon that originates a truncated cytoplasmic tail, consequently leading to the loss of intracellular protein domains and signalling motifs necessary for interaction with cytoskeletal components.
- **CD44s-exon15** A CD44s homolog of 37 kDa lacking the peptide sequence encoded by exon 15.
- **CD44sol** This CD44 soluble isoform only retains exons 1–4, while presenting truncated forms of the exons 3 and 4. The modification of the two later exons leads to a smaller extracellular domain as well as to the loss of transmembrane and cytoplasmic domains. This 16 kDa isoform is often shed to bodily fluids through matrix metalloprotease activity.

In summary, the wide array of structurally similar CD44 isoforms, associated with dense glycosylation and inadvertent lack of nomenclature consensus has posed a significant challenge for inferring on CD44 role in cancer. These aspects have biased many previous conclusions, provided several conflicting data, and significantly delayed clinical development. Most studies disregard CD44 glycosylated domains, which sometimes more than double the molecular weight of the isoforms, decisively modulating biophysical, biochemical and functional properties of the receptor (predicted glycosylation sites detailed in Table 1). Glycosylation also raises a tremendous challenge for CD44 mapping based on conventional proteomics, urging the introduction of glycan-targeted approaches. As such, more comprehensive strategies will certainly require the integration of glycomics/glycoproteomics with emerging proteogenomics, exploring customized protein sequence databases generated using genomics and transcriptomics. This approach is also expected to pinpoint relevant cancer neoantigens for driving targeted therapeutics and immunotherapy development. Nevertheless, we augment that the necessary technologies are now available for addressing CD44 molecular and functional diversity in health and disease, ultimately providing targetable biomarkers for oncology.

## Abbreviations

CD44: cluster of differentiation 44
HCELL: hematopoietic cell E-/L-selectin ligand
RNAseq: RNA sequencing
mRNA: messenger ribonucleic acid
